# Integrative transcriptomic and metabolomic analyses provide insights into the mechanism of autotoxicity of *Pugionium cornutum* (L.) Gaertn

**DOI:** 10.1371/journal.pone.0331858

**Published:** 2025-09-17

**Authors:** Kezhen Ning, Fenglan Zhang, Zhongren Yang, Xiaoyan Zhang, Dong Zhang, Xinyuan Qin, Xiumei Huang, Lizhen Hao

**Affiliations:** 1 College of Horticultural and Plant Protection, Inner Mongolia Agricultural University, Inner Mongolia Key Laboratory of Wild Peculiar Vegetable Germplasm Resource and Germplasm Enhancement, Huhhot, China; 2 Inner Mongolia Autonomous Region Key Laboratory of Big Data Research and Application for Agriculture and Animal Husbandry, Hohhot, China; University of California Riverside, UNITED STATES OF AMERICA

## Abstract

*Pugionium cornutum* (L.) Gaertn., a functional vegetable with dual health benefits, faces significant constraints in sustainable industrial development due to autotoxicity-induced continuous cropping obstacles. This study investigates the molecular regulatory mechanisms underlying the response of *Pugionium cornutum* (L.) Gaertn. to phthalic acid, a representative autotoxic substance, through integrated transcriptomic and metabolomic analyses under varying phthalic acid concentrations. The results revealed that phthalic acid stress significantly altered the abundance of 892 metabolites. Critical metabolites including MG(16:0/0:0/0:0)[rac] and 6-hydroxysphingosine were found to modulate differentially expressed genes (DEGs) involved in key pathways such as flavonoid biosynthesis, tropane/piperidine/pyridine alkaloid biosynthesis, glucosinolate metabolism, and ABC transporter activity. Comparative analysis demonstrated more pronounced molecular responses under high-concentration phthalic acid stress (10 mmol/L) compared to low-concentration treatment (0.1 m mmol/L), indicating intensified phytotoxic effects at elevated autotoxin levels. These findings provide novel insights into the autotoxicity response mechanisms of *Pugionium cornutum* (L.) Gaertn.and establish a theoretical foundation for developing sustainable cultivation strategies for this species.

## Introduction

*Pugionium cornutum* (L.) Gaertn. is a plant endemic to China (a biennial herb), which is extensively applied in soil erosion control because of its robust root system and potent sand-fixing capacity [1]. In addition, this plant has substantial medicinal properties and is a wholesome, nutritious green vegetable with a high protein content and low sugar level [2,3]. However, the species has been on the verge of extinction in recent years mainly because the plant cannot be cropped continuously. Specifically, several years of continuous cultivation results in continuous cropping obstacles of *Pugionium cornutum* (L.) Gaertn., which reduces its quality and yields, a phenomenon that is also called autotoxicity [4]. Autotoxicity, a type of abiotic stress, results in poor growth, high pest and disease incidence, reduced crop quality, and decreased yields. Numerous plant species, including weeds and crops, have shown evidence of this phenomena [5,6]. Plants produce specific stress responses when subjected to biotic or abiotic stresses, affecting gene expression [7,8], ultimately altering metabolites. Therefore, analyzing the relevant pathways and metabolites in plants is of great significance for studying the mechanisms of autotoxicity.

Plant metabolomics is defined as qualitative and quantitative analyses of metabolite compositions and the study of their metabolic networks to explain the relevant phenotypes. The plant metabolome includes both primary metabolites necessary for plant growth and development, such as organic acids, lipid molecules, and carbohydrates, and secondary metabolites closely related to plant stress resistance, such as flavones, alkaloids, phenols, and lignin. Peng et al [9] investigated the changes in metabolites in rice varieties after stress by metabolomics and found that the metabolite contents of different metabolic pathways differed, and the activation of the gene BPH15 increased the content of secondary metabolites, which in turn enhanced the resistance of rice.Eloh et al. [10] used metabolomics techniques to analyze changes in metabolites in tomato leaves and stems before and after nematode infestation, which demonstrated obvious upregulation of β-alanine and phenylalanine and significant downregulation of glycerol and ribose in leaves, as well as induction of glucose and sucrose and downregulation of fumaric acid and glycine in stems. Autotoxic substances, which are primarily produced through root exudates and residues, are key contributors to the occurrence of autotoxicity. Therefore, the most significant factor causing transplanting disorders and encouraging the alteration of the microbial community is the buildup of self-toxic chemicals in the soil [11]. Terpenoids, steroids, phenols, coumarins, flavonoids, tannins, alkaloids, and other groups of chemicals have all been identified as autotoxic. Among these, phenolic compounds have been the most extensively investigated for their phytotoxicity [12,13]. Numerous physiological processes and metabolic systems in plants can be significantly disrupted by autotoxins [14]. It has been shown that self-toxicant stress can alleviate the self-toxicity of cucumber seedlings by regulating membrane lipid peroxidation and improving photosynthetic efficiency [15]. The root system of melon has a dynamic response mechanism to self-toxicity, and the HCT gene and CYP gene show significant expression changes under self-toxicity [16].

Plants are frequently exposed to various abiotic and biotic stresses that hinder their growth and development. To cope with these adverse conditions, plants initiate complex adaptive responses at multiple levels, including molecular, cellular, organ, physiological, and biochemical processes. However, the underlying molecular mechanisms governing these responses remain incompletely understood due to their intricacy. Advances in molecular biology and bioinformatics have accelerated research on plant stress responses, shifting the focus from single-omics analyses to more integrated systems biology approaches. In particular, metabolites not only reflect gene expression but also serve as the direct chemical basis of physiological states. Given the nonlinear and dynamic nature of plant responses, multi-omics strategies offer a more comprehensive and in-depth understanding of plant adaptation mechanisms.In order to mimic the autotoxicity phenomena in plants, phthalic acid was applied to *Pugionium cornutum* (L.) Gaertn. seedlings in this investigation, and then RNA was extracted from phthalic acid-treated roots to construct an RNA-sequencing (RNA-seq) library. Through the identification of enriched functional items in response to autotoxicity and a thorough clarification of the roots’ reaction to phthalic acid stress, this study laid the groundwork for increasing *Pugionium cornutum* (L.) Gaertn.’s resistance or tolerance to environmental autotoxins.

## Materials and methods

### Ethics statements

The plant materials used in this study were collected in accordance with local biodiversity regulations andcultivated inauthorized botanical repositories. Following the ethical guidelines of the InternationalSociety of Ethnobiology (2008) andChina’s Regulations on the Protection of Wild Plants (2022), noadditional ethical approval was required fornon-endangered plant species used exclusively for molecularanalyses.

### Experimental materials and stress treatment

**Fig 1:**
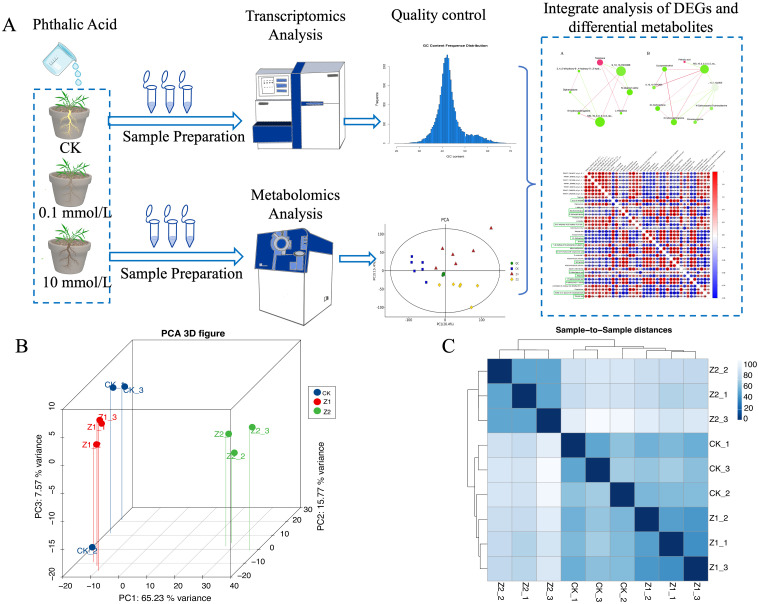
Experimental design and sample relationship analysis. (A) Schematic diagram of experimental design. (B) Plot of principal component analysis between the treated and control samples. (C) Heat map of inter-sample correlation.

The experiment was carried out in an intelligent greenhouse (integrates sensing and control systems) at Inner Mongolia Agricultural University. The plant materials used had been formally identified by our group in a previous study, and *Pugionium cornutum* (L.) Gaertn. [1] were stored in the laboratory. For the experiment, we chose seeds that were completely mature. After sterilizing the seeds with 2% sodium hypochlorite, to start germination, they were placed in a light incubator that was set at 28°C. Seeds that germinated consistently were sown in pots containing a mixture of vermiculite and soil (1:1 by volume) (Fig 1A). When the cotyledons have fully unfolded (15 days after germination), the treatment begins. Plants in the control condition (CK) were irrigated with distilled water, whereas the treatment group was administered phthalic acid solutions at concentrations of 0.1 mmol/L (Z1) and 10 mmol/L (Z2)(100 mL per pot per time) at 3-day intervals (Fig 1A). Phthalic acid (Sinopharm Chemical Reagent Co., Ltd., Shanghai, China) was dissolved in preheated distilled water to prepare 0.1 mmol/L and 10 mmol/L solutions. Solutions were stirred until fully dissolved, cooled to room temperature, and used immediately. When the seedlings reached the six-leaf stage and had developed a bud, root samples were collected for transcriptomic and metabolomic analyses. For each treatment, four independent experiments were conducted. Metabolomic analysis was performed using six biological replicates per treatment, while transcriptome sequencing was performed using three biological replicates.

### Metabolite extraction from samples

All chemicals and solvents used in the analysis were of analytical or HPLC grade. Acetonitrile, methanol, water, and formic acid were purchased from Thermo Fisher Scientific (Waltham, MA, USA). L-2-chlorophenylalanine was obtained from Shanghai Hengchuang Biotechnology Co., Ltd. (Shanghai, China). For sample preparation, 80 mg of freeze-dried material was accurately weighed into a 1.5 mL Eppendorf tube, followed by the addition of two 3 mm diameter chrome steel balls. After being combined with 1 mL of a methanol and water (7/3, vol/vol) mixture and 20 μL of L-2-chlorophenylalanine (0.3 mg/mL) dispersed in methanol as an internal standard, the samples were subjected to 30 minutes of ultrasonic extraction in an ice-water bath, 20 minutes at −20°C, and 2 minutes of grinding at 60 Hz. They were then stored at −20°C for two minutes. Following a 10-minute centrifugation at 4°C (13,000 rpm), the samples were passed through microfilters with a pore size of 0.22 μm and moved to LC vials. Next, 150 μL of the supernatants from each tube were decanted using crystal syringes. For QC, a pooled sample was made by combining an aliquot of each sample.

### LC-MS sample up-processing

The Nexera UPLC system (Shimadzu, Japan) was coupled with a Q Exactive quadrupole–orbitrap mass spectrometer equipped with a heated electrospray ionization (HESI) source (Thermo Fisher Scientific, Waltham, MA, USA). Metabolic profiling was performed in both positive and negative ESI ionization modes. Chromatographic separation was achieved using an ACQUITY UPLC HSS T3 column (1.8 μm, 2.1 × 100 mm) under a binary gradient elution protocol. Flow phase A consisted of 95% water and 5% acetonitrile (containing 0.1% formic acid), while phase B consisted of 47.5% acetonitrile, 47.5% isopropanol, and 5% water (containing 0.1% formic acid). The flow rate was set at 0.40 mL/min, and the column temperature was maintained at 40°C. All samples were maintained at 4°C throughout the analysis. The mass scan range was set to m/z 125–1000, with a resolution of 70,000 for full MS scans. The collision energies were set to 10, 20, and 40 eV. The capillary temperature was maintained at 320°C. Quality control (QC) samples were injected at regular intervals throughout the run to monitor system stability and ensure data reproducibility.

### RNA extraction and detection

Total RNA was extracted from plant root samples using the mirVana™ miRNA Isolation Kit (Ambion, Naugatuck, CT, USA) following the manufacturer’s instructions. The extracted RNA was eluted in 100 μL of RNase-free water preheated to 95°C and immediately stored at −80°C. RNA concentration and purity were assessed using a NanoDrop 2000 spectrophotometer (Thermo Fisher Scientific, USA), and RNA integrity was confirmed by 0.1% agarose gel electrophoresis.

### cDNA library construction

Using an Agilent 2100 Bioanalyzer (Agilent Technologies, Santa Clara, CA, USA), the RNA’s integrity was evaluated. For further examination, samples having an RNA integrity number (RIN) ≥ 7 were chosen. Following the manufacturer’s instructions, the libraries were constructed using the TruSeq Stranded mRNA LT Sample Preparation Kit (Illumina, San Diego, CA, USA). The Il-lumina sequencing technology (HiSeq TM 2000) was then used to sequence nine libraries, producing paired-end reads (150 bp) (http://www.biomarker.com.cn/technology-services/medicine-ont-quanchang).

### Data processing and differential analysis

Progenesis QI V2.3 software (Nonlinear, Dynamics, Newcastle, UK) was used to handle raw LC-MS data for both qualitative and quantitative analysis. The positive and negative data were combined to get a combine data which was imported into R ropls package. Principle component analysis (PCA) and (orthogonal) partial least-squares-discriminant analysis (O)PLS-DA were carried out to visualize the metabolic alterations among experimental groups, after mean centering (Ctr) and Pareto variance (Par) scaling, respectively. The Hotelling’s T2 region, shown as an ellipse in score plots of the models, defines the 95% confidence interval of the modeled variation. Variable importance in the projection (VIP) ranks the overall contribution of each variable to the OPLS-DA model, and those variables with VIP > 1 are considered relevant for group discrimination.

The differential metabolites were selected on the basis of the combination of a statistically significant threshold of variable influence on projection (VIP) values obtained from the OPLS- DA model and p values from a two-tailed Student’s t test on the normalized peak areas, where metabolites with VIP values larger than 1.0 and p values less than 0.05 were considered as differential metabolites.

### RNA-seq data analysis

RNA sequencing and preliminary analysis were conducted by OE Biotech Co., Ltd. (Shanghai, China). Raw reads in FASTQ format were filtered using Trimmomatic [17] to remove adapters, low-quality bases, and ambiguous nucleotides (N), resulting in high-quality clean reads, are provided in S2 Table. Clean reads were assembled into transcripts using Trinity (v2.4) [18] with the paired-end method. The longest transcript in each cluster was selected as a unigene.

Functional annotation of unigenes was performed using BLASTx [19] (E-value < 1e-5) against the COG, and SwissProt databases. Based on SwissProt results, GO terms were assigned, and pathway annotations were obtained by aligning unigenes to the KEGG database [20].

Gene expression levels (FPKM and raw counts) were calculated using Bowtie2 [21] and eXpress [22]. Read counts were normalized using the estimateSizeFactors function of the DESeq R package [23], and differential expression analysis was conducted with the nbinomTest function. Unigenes with p < 0.05 and fold change > 2 were considered significantly differentially expressed. GO and KEGG enrichment analyses were performed to determine the biological functions and pathways associated with differentially expressed genes. Hierarchical clustering and heatmaps were used to visualize expression patterns. The RNA-seq data generated in this study are not publicly available but are available from the corresponding author upon reasonable request.

## Result

### PCA of transcriptome data and inter-sample correlation analysis after exogenous phthalic acid treatment

Transcriptome sequencing was performed after the samples were exogenized with Z1 and Z2, respectively. PCA results showed that there were significant differences between control samples (CK_1, CK_2 and CK_3) and treatment samples (Z1_1, Z1_2 and Z1_3, and Z2_1, Z2_2 and Z2_3), with relatively favorable inter-sample differentiation, which indicated that the transcriptome data for sequencing matched sequencing requirements (Fig 1B). Meanwhile, the samples’ correlation showed that the samples treated with Z1 had a stronger connection with the untreated samples than the ones treated with Z2. (Fig 1C).

#### Statistical analysis of differential genes in the transcriptome.

**Fig 2:**
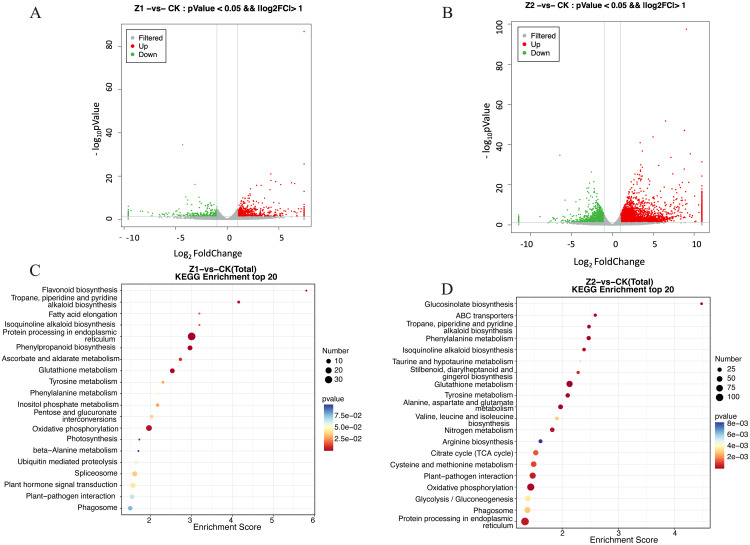
Differential gene volcano map and KEGG enrichment pathway. (A)Volcano maps of differential genes after exogenous treatment with Z1. (B)Volcano maps of differential genes after exogenous treatment with Z2.(C)The pathway of KEGG enrichment under Z1 treatment is shown, highlighting the specific biological processes affected by the low concentration. (D)The pathway of KEGG enrichment under Z2 treatment is shown, highlighting the effects on biological processes at high concentrations.

Gene expression was analyzed differently following exogenous treatment with Z1 and Z2, respectively. The data showed that a total of 1,085 genes were altered in the samples treated with Z1 as compared to the control samples, including 703 upregulated genes and 382 downregulated genes (Fig 2A). Likewise, as phthalic acid concentration increased, the number of differential genes increased to 5843, with 4023 genes showing significant up-regulation and 1820 showing significant down-regulation in the Z2-treated samples (Fig 2A). The results illustrated that exogenous treatment with Z2 resulted in more pronounced gene changes and had a greater effect on the plants.

### KEGG analysis of differential genes

KEGG enrichment analyses were performed on the differential genes in the samples exogenously treated with Z1 and Z2, respectively.The results presented that the differential genes in the samples exogenously treated with Z1 were mainly enriched in pathways including Plant hormone signal transduction, Flavonoid biosynthesis, Tropane, piperidine and pyridine alkaloid biosynthesis, Oxidative phosphorylation, with the most significant enrichment in pathways including Glutathione metabolism and other processes (Fig 2C).And most the highest number of differential genes enriched in Plant hormone signal transduction, Oxidative phosphorylation. Two of the most enriched pathways are the Flavonoid biosynthesis and Tropane, piperidine and pyridine alkaloid biosynthesis.

Similarly, we found that the following pathways were significantly altered by Z2 treatment, the Glutathione metabolism, ABC transporters, Phenylalanine metabolism, and Oxidative phosphorylation, Plant-pathogen interaction and other processes. And the greatest quantity of genes that differ were enriched in pathways such as Protein processing in endoplasmic reticulu, Oxidative phosphorylation (Fig 2D). Among the pathways significantly enriched under Z2 treatment were Glucosinolate biosynthesis and ABC transporters. These results highlighted that exogenous phthalic acid treatment affected the internal metabolic changes in plants, with the exogenous treatment of Z2 exerting a greater effect. These findings demonstrate that exogenous phthalic acid treatment can significantly influence the internal metabolic processes of plants. Among the treatments, the application of 10 mmol/L phthalic acid resulted in the most substantial changes, affecting a greater number of genes and exhibiting a more pronounced impact.

**Fig 3:**
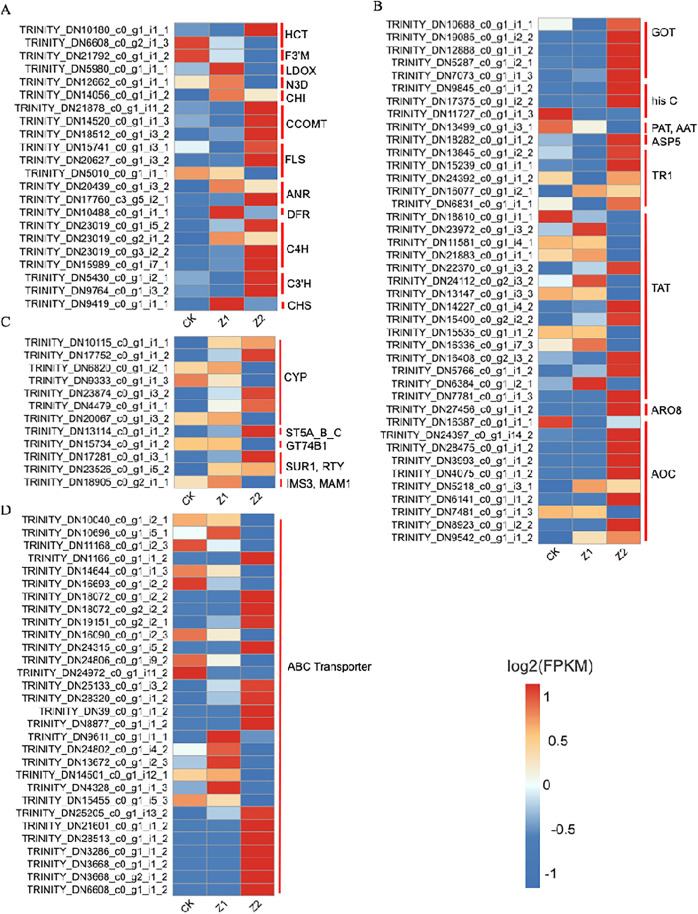
Heatmap showing the expression profiles of differential genes involved in phthalic acid stress. (A) Differential genes involved in Flavonoid biosynthesis. (B) Differential genes involved in Tropane, piperidine and pyridine alkaloid biosynthesis. (C) Differential genes associated with the metabolic pathway of Glucosinolate biosynthesis. (D) Differential genes associated with ABC transporters. The colours of the heatmap cells indicate the proportion of gene expression levels in different samples. From blue to red indicates a gradual increase in gene expression levels.

We next analysed the first 2 pathways enriched under different concentration treatments of phthalic acid. In this study, a large number of differential genes associated with the stress response were significantly regulated by phthalic acid stress. Phthalic acid significantly induced shikimate O-hydroxycinnamoyltransferase (HCT), flavonoid 3’-monooxygenase (F3’M), leucoanthocyanidin dioxygenase (LDOX), naringenin 3-dioxygenase (N3D), chalcone isomerase (CHI), caffeoyl-CoA O-methyltransferase (CCOMT), flavonol synthase (FLS), anthocyanidin reductase (ANR), bifunctional dihydroflavonol 4-reductase (DFR), trans-cinnamate 4-monooxygenase (C4H), coumaroylquinate(coumaroylshikimate) 3’-monooxygenase (C3’H), chalcone synthase (CHS)expression level (Fig 3A). Under phthalic acid treatment, there were 5 aspartate aminotransferase (GOT), 3 histidinol-phosphate aminotransferase (hisC), 1 bifunctional aspartate aminotransferase and glutamate/aspartate-prephenate aminotransferase (PAT, AAT), 1 aspartate aminotransferase, chloroplastic (ASP5), 5 tropinone reductase I (TR1), 15 tyrosine aminotransferase (TAT), 1 aromatic amino acid aminotransferase I (ARO8)and 10 primary-amine oxidase (AOC) (Fig 3B).The Glucosinolate biosynthesis metabolic pathway was least differentially characterized after phthalic acid treatment, with a total of 12 differentially characterized genes (Fig 3C). The expression levels of 30 ABC transporters genes were also significantly induced after phthalic acid treatment (Fig 3D). Interestingly, the expression of most of these stress-responsive genes showed an upward trend under high concentration of phthalic acid treatment. It suggests that they play an important role in the response of sand mustard to high concentration of phthalic acid stress.

### Co-expression network analysis of differential genes

**Fig 4:**
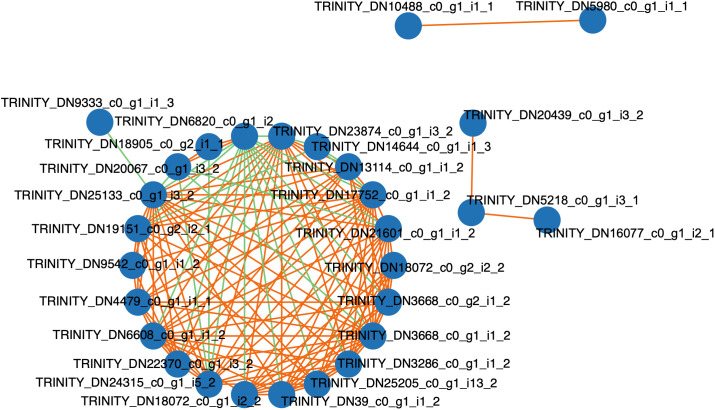
Co-expression map of differential genes (Orange is positively correlated and green is negatively correlated).

KEGG enrichment analyses were conducted on the differential genes after exogenous treatment with Z1 and Z2, respectively, which identified key metabolic pathways, such as phenylalanine metabolism, plant-pathogen interactions, and glutathione metabolism. Differential genes in these several highly related pathways were subjected to co-expression network analysis, which revealed that these genes had significant co-expression relationships and might be synergistically involved in plant autotoxicity (Fig 4). Among them, TRINITY_DN3668_c0_g1_i1_2 (connectivity 21), TRINITY_DN17752_c0_g1_i1_2 (connectivity 19) and TRINITY_DN6820_c0_g1_i2_1 (connectivity 16)are the core regulator nodes in the network. TRINITY_DN3668_c0_g1_i1_2 is described as ABC transporter. The function of TRINITY_DN17752_c0_g1_i1_2 has been described as being aromatic aldoxime N-monooxygenase. Among them, TRINITY_DN6820_c0_g1_i2_1 had 14 highly negatively correlated connections, and the functional compartment (methylthio)alkanaldoxime N-monooxygenase may maintain the network dynamic equilibrium through negative regulation.

### PCA of metabolomic data

**Fig 5:**
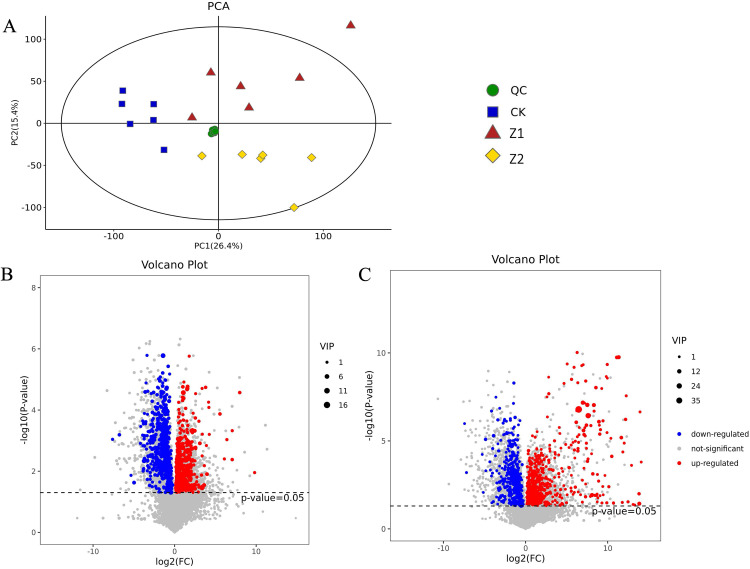
PCA analysis of metabolites between different treatment groups and volcano plot analysis of different metabolites. (A) Plot of the samples’ PCA scores from groups Z1, Z2 and CK. (B) Differential metabolite volcanic plots in Z1 and CK. (C) Differential metabolite volcanic plots in Z2 and CK. Note: In the (A) plots, the horizontal coordinate is the logarithm of the multiplicity of differences in the relative levels of the two groups of metabolites (log2FC), with larger absolute values indicating greater differences. Each point in the (B) and(C)plots represents a metabolite, with blue representing down-regulated metabolites, red representing up-regulated metabolites, and gray indicating de-regulated metabolites with insignificant differences.

In addition to transcriptome sequencing, metabolome sequencing was also carried out to ascertain the effects of exogenous phthalic acid treatment on plant metabolism. As depicted in Fig 5A, significant dispersion occurred between treatments but not within groups, showing a significant change in the metabolites in the samples following various treatments. Further illustration of the reliability and consistency of metabolomics data.

### Metabolite-related network analysis

A combination of FC and VIP values was used to screen for differentiating metabolites. Sequencing results showed a total of 892 different metabolites (S1 Table). There were 331 metabolites up-regulated and 126 metabolites down-regulated under Z1 treatment (Fig 5B). Under Z2 treatment (Fig 5C) 306 metabolites were up-regulated and 129 metabolites were down-regulated. It can be clearly concluded that differential metabolites were fewer with less abundance in the samples treated with Z2 than in the samples treated with Z1.

**Fig 6:**
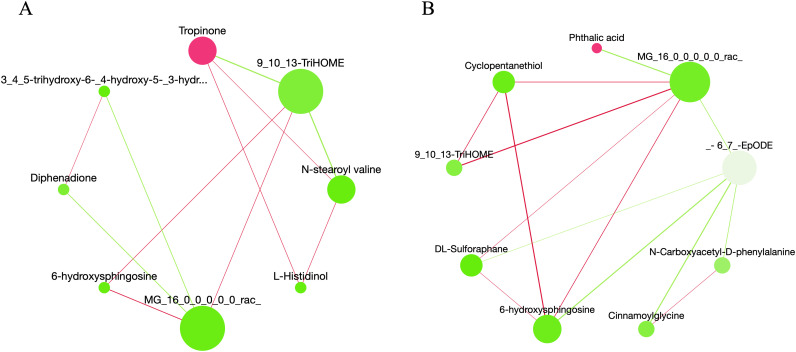
Metabolite related network diagrams. (A) Correlation networks of differential metabolites under Z1 and CK treatment. (B) Correlation network of differential metabolites under Z2 and CK treatment. The correlation network diagram uses sum-abundance values to represent the nodes, with green denoting a low value and rosy red a high value; a rosy line indicates a positive correlation, a green line denotes a negative correlation, and the line thickness indicates the correlation’s strength.

The difference metabolites identified in the two comparison groups were subjected to network correlation analysis. In Z1-CK (Fig 6A), 9,10,13-TriHOME exhibited negative correlations with Tropinone and N-stearoyl valine, while showing positive correlations with MG(16:0/0:0/0:0)[rac] (MAG) and 6-hydroxysphingosine (6-HSph). Additionally, MG was positively correlated with both 9,10,13-TriHOME and 6-HSph. In Z2-CK (Fig 6B), MAG was positively correlated with Cyclopentanethiol, while 9,10,13-TriHOME, DL-Sulforaphane, and 6-hydroxysphingosine were all positively correlated with phthalic acid. Conversely, −6(7)-EpODE (cis-6(7)-Epoxy-9-hydroxy-10,12,15-octadecatrienoic acid) exhibited a negative correlation. A positive correlation was observed between metabolites MAG and 6-HSph in both treatments. Among them, MG and (6-HSph) are lipids, and lipids play a role in plant response to various stresses [24]. Plants can adapt to environmental changes by altering lipid metabolism [25,26].

### Exogenous phthalic acid treatment induces changes in gene expression, thus consequently altering the type and level of metabolites

**Fig 7:**
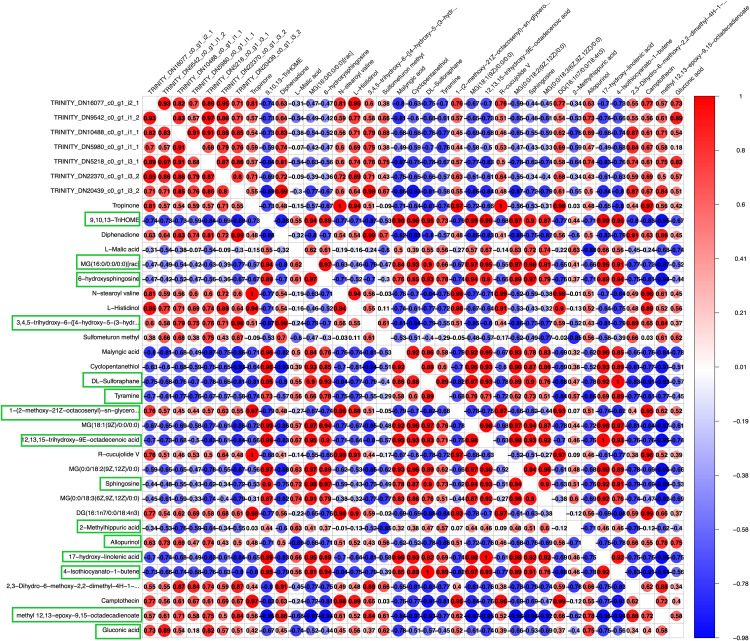
Map of correlation between differential genes and metabolites. Green boxes indicate metabolites that are co-enriched under both Z1 and Z2 treatments. Positive correlation in red, negative correlation in blue.

To demonstrate the regulatory features between genes and metabolites, correlation analysis for differential genes and differential metabolites were performed (Fig 7 and S1 Fig). Genes within the highly correlated pathway and metabolites from the top 30 were analyzed for their associations. Fifteen metabolites were found to be co-enriched under the Z1 and Z2 treatments. Specifically, under the Z1 treatment (Fig 7), five metabolites exhibited positive correlations with the corresponding genes, while ten showed negative correlations. We found that the metabolites MAG and 6-HSph behaved consistently and were negatively correlated with 7 genes enriched in the Flavonoid biosynthesis pathway and the Tropane, piperidine and pyridine alkaloid biosynthesis pathway. Previous studies have shown that the gene expression levels of these seven differential genes were significantly elevated under Z1 treatment, implying that MAG and 6-HSph negatively regulate low concentrations of phthalic acid stress. Suggests that they are closely related to the Flavonoid biosynthesis pathway and the Tropane, piperidine and pyridine alkaloid biosynthesis pathway

In contrast, under the Z2 treatment (S1 Fig), the majority of metabolites displayed negative correlation with the associated genes, with only a few showing positive correlations. Among them, MAG and 6-HSph were positively correlated with 5 differential genes and 17 differential genes were said to be negatively correlated. Unlike the low phthalic acid treatment, MAG and 6-HSph were involved in both positive and negative regulation under high phthalic acid treatment. This implies that they are closely related to the Glucosinolate biosynthesis pathway and the ABC transporters pathway under high phthalic acid treatments and the degree of response becomes complex. This suggests a greater degree of variation in the autotoxic response of *Pugionium cornutum* (L.) Gaertn. to high concentrations of phthalic acid.

These results further substantiate that the differential expression of genes mediated by phthalic acid is strongly correlated with the respective metabolites, demonstrating their critical function in the plant’s phthalic acid autotoxicity response. Differential response of *Pugionium cornutum* (L.) Gaertn. to different concentrations of phthalic acid

## Discussion

Autotoxicity is a form of allelopathy, manifested internally [27,28], where it affects plant growth through mechanisms such as root secretion [29,30]. As a significant ecological chemical factor, autotoxicity is essential for preventing plant growth [31,32]. Therefore, it is crucial to clarify *Pugionium cornutum* (L.) Gaertn.‘s autotoxic pathways in order to overcome the plant’s ongoing cropping challenges. Meng et al. found that stress induced by the allelopathic compound cinnamic acid (CA) significantly reduces the activity of key enzymes in plants [33]. Han et al. further revealed that benzoic acid, even at low concentrations, can trigger autotoxicity effects in plants [34,35]. Wang, R., and colleagues, using integrated transcriptomic and metabolomic analyses, elucidated that ginseng can accumulate metabolites that disrupt the regulatory responses of related genes, thereby inducing autotoxicity mechanism [36]. Furthermore, further research has demonstrated that ginsenosides control the expression of genes encoding important enzymes engaged in vital metabolic pathways, hence producing allelopathic autotoxicity effects [37]. These findings collectively suggest that plants under toxic stress release allelochemicals through various mechanisms, thereby modulating internal processes and ultimately affecting their growth performance [38]. In this study, *Pugionium cornutum* (L.) Gaertn. roots exogenously treated with phthalic acid were the subject of transcriptome and metabolomics investigations in this study. It is commonly known that transcriptional modifications in gene expression are essential for plants to adapt to stressful environments. Stress-responsive genes are activated, stress-related chemicals and their metabolic pathways are regulated, and plants undergo a range of physiological and molecular changes in response to extreme environmental stressors [39,40]. Previous studies have demonstrated that abiotic stress directly impacts the activity of key enzymes and the expression of genes involved in plant metabolism [41]. Furthermore, it has been demonstrated that depending on the dosage and length of treatment, plants react differently to autotoxic substances as 2,4-DTBP [32,42]. In our study, we observed that the extent of gene expression changes varied with different concentrations of phthalic acid treatments. Notably, higher concentrations induced more pronounced gene alterations, exerting greater effects on the plants. These findings are consistent with previous studies [15,36], further confirming that phthalic acid induces significant changes in the expression of key genes. This highlights a complex regulatory mechanism in *Pugionium cornutum* (L.) Gaertn. under the stress of varying concentrations of autotoxic substances. Similar to the present study, the synthesis of secondary metabolites was closely related to stress response, but the flavonoid and alkaloid pathways of sand mustard were enriched at low concentrations of PHTHALIC ACID, whereas ginseng was more dependent on the accumulation of saponins [36], suggesting that different crops may cope with autotoxic stress through different secondary metabolites [43]. In contrast, *Pugionium cornutum* (L.) Gaertn. mitigated oxidative damage through flavonoids (antioxidant function) at low PHTHALIC ACID concentrations, while shifting to thioglycosides (de-fense compounds) and ABC transporter proteins (detoxification function) at high concentrations, suggesting that plants may adjust their metabolic strategies according to stress intensity [44]. In this study, we found a significant enrichment of ABC transporter protein genes in Pugionium cornutum (L.) Gaertn. under high concentrations of phthalic acid, suggesting that the ABC family may have a conserved function in the efflux of self-toxicants from a variety of crops. However, ABC genes in Pugionium cornutum (L.) Gaertn. may respond specifically to phthalic acid stress. In contrast, the ABC family in rice is more biased toward the transport of phenolic acids, suggesting functional differentiation of different transporter protein families. The autotoxic effects of ginsenosides are associated with hormone signaling pathways regulated by genes such as JAR1 and COI1 [36]. In contrast, Pugionium cornutum (L.) Gaertn. does not involve hormonal pathways and may be directly involved in membrane lipid stabilization or signaling through metabolites (MAG, 6-HSph), reflecting the diversity of detoxification mechanisms. In this study, Pugionium cornutum (L.) Gaertn. was found to be more responsive to high concentrations of phthalic acid, as evidenced by an increase in the number of differential genes. This is consistent with studies on melon: melon growth is inhibited under long-term self-poisoning stress and significant expression changes in differential genes occur under self-poisoning [16,45]. In addition, soybean induces more differential genes and metabolites (e.g., flavonoids) under drought stress with high PEG treatments [46]. These results suggest that increases in concentration or stress intensity may generally trigger more complex defense responses in plants, but their specific regulatory networks vary by species and stress type. Our study suggests that MAG and 6-HSph are involved in phthalic acid stress response, which is inconsistent with the finding of metabolites involved in autotoxicity in soybean [46] and cucumis melo [45]. This could be due to different plants. However, the role of its specific lipid metabolites is reported for the first time in self-toxicity studies, providing new evidence for the association of plant lipid metabolism with self-toxicity mechanisms.

Zhang et al. investigated tomato fruits that accumulated various flavonoids and discovered a favorable relationship between the tomatoes’ resistance to Botrytis cinerea and the accumulation of particular flavonoids [47]. Flavonoids are also associated with photosynthesis in plants, and lower red/far-red ratios of the light reduce the content of flavonoids such as anthocyanidin and kaempferol in tomato stems, which severely affects the capacity of the plant for photosynthesis [48]. In a study on rice, naringenin and sakuranetin were found to be effective against bacterial and fungal pathogens, respectively, and were able to provide protection against different pathogens in different rice-growing environments by adjusting flavonoid contents [49]. BPH is a rice-specific pest. A prior study unraveled that overexpression of OsGRF8 increased flavonoid contents in rice, which elevated resistance to BPH, and that exogenous application of flavonoids also enhanced resistance to BPH in wild-type varieties [50]. Zhang et al. [51] used CRISPR/Cas9-mediated multiplex gene-editing technology to create T0 transgenic soya bean plants for metabolomic research and noticed that the concentration of isoflavones was dramatically enhanced, which facilitated the resistance of leaves to soya bean mosaic viruses. The autotoxicity of ginseng leads to continuous cropping obstacles. The pathways through which ginseng induces allelopathic autotoxicity differ from those of *Pugionium cornutum* (L.) Gaertn. This discrepancy may be due to the differences between the plant species [5,37,52]. Previous studies have confirmed that continuous cropping reduces bacterial species diversity, alters bacterial community structures, increases harmful microorganisms, and decreases beneficial microorganisms [53,54]. However, further investigation is required to comprehend the alterations that take place in bacteria following phthalic acid treatment. Additionally, abscisic acid is also an essential phytohormone for plant development, growth, and stress tolerance, Downregulation of the gene encoding this enzyme may result in reduced synthesis of abscisic acid, impairing the plant’s ability to resist biotic and abiotic stresses [55]. Enhanced resistance to fungal infections is demonstrated by transgenic tobacco plants that carry the defensin gene (RS-AFP1) [56], while the defensin gene product (BSD1) demonstrates antifungal action against a variety of phyto-pathogenic fungi [57]. Plant defense strategies include the accumulation of glycine-rich proteins in vascular tissues [58]. Therefore, decreased resistance to phytopathogenic fungi and impaired defensive mechanisms result from the downregulation of genes encoding glycine-rich proteins, defensin-like proteins, and those involved in biostimulation responses. This has a detrimental effect on seedling growth.

In the presence of biotic or abiotic stresses, specific stress responses occur in plants, ultimately triggering changes in metabolites. Plant metabolomics refers to qualitative and quantitative analyses of metabolite compositions and the study of their metabolic net-works to explain the relevant phenotypes. The plant metabolome includes both primary metabolites responsible for plant growth and development, such as organic acids, lipid molecules, and carbohydrates, and secondary metabolites closely correlated with plant stress resistance, such as flavones, alkaloids, phenols, and lignin. Secondary metabolites produced in plants not only contribute to resistance to adverse conditions such as pests and diseases, droughts, and flooding but also are involved in the maintenance of plant ho-meostasis [59]. In this investigation, 892 distinct metabolites were found in the samples, and these metabolites, which primarily included flavonoid production, varied significantly between groups. Of note, flavonoid metabolites play a pivotal role in the stress resistance of plants. A total of 457 differential metabolites were screened in the Z1/CK group (S1 Table), with upregulated 331 metabolites, including 54 flavonoids and 12 alkaloids. A total of 435 differential metabolites were yielded in the Z2/CK group, with 306 upregulated metabolites, including 54 flavonoids and 12 alkaloids. These findings in-dicated that these metabolites were important for the occurrence of autotoxicity in plants. In this study, it was shown that MAG and 6-HSph are key metabolites in the regulation of phthalic acid autotoxicity. MAG is a monoacylglycerol, plays an important role in plant growth and development, especially in lipid metabolism, root development, and root secretions. Lipids are important components of plant cells and are involved in a variety of physiological processes such as energy storage, membrane structure construction and signal transduction. Changes in the levels of MAG, an intermediate product of lipid metabolism, may affect plant growth [60]. It has been shown that Mg deficiency affects lipid metabolism in plants, which in turn affects plant growth [41]. In addition, MAG, as an intermediate product of lipid metabolism, may be involved in the regulation of plant growth and development through a variety of pathways affecting lipid metabolism, root development, and root secretions [24]. Under stressful environments, plants alter lipid metabolism to adapt to environmental changes. For example, under salt stress, plants may maintain membrane stability by changing the composition of membrane lipids [61].Plant sphingolipids are involved in cell signaling, maintenance of membrane structure and response to environmental stresses [62,63]. They play the role of signaling molecules in plant response to biotic and abiotic stresses [64,65]. Studies have shown that sphingolipid homeostasis regulation is critical for balancing plant life and death [26,66]. Sphingolipids are involved in plant cell death and defense responses and are essential for plant immunity. As a key molecule in sphingolipid metabolism, 6-HSph plays a complex and important role in plant response to various stresses by participating in cell signaling, gene expression regulation, and interactions with other plant hormones and metabolites. Stressful environments affect the biosynthesis of plant secondary metabolites, which is closely linked to transcriptional regulation [67].Overall, in the presence of external environmental stress, plants may elevate their energy metabolism by regulating the levels of sugars and amino acids to resist the effects of the external environment.

## Conclusions

In this study, we used a combination of transcriptomic and metabolomic analyses to explore the response of *Pugionium cor-nutum* (L.) Gaertn. to different concentrations of phthalic acid treatments. Phthalic acid treatment resulted in significant changes in gene expression and metabolic levels of *Pugionium cornutum* (L.) Gaertn. We observed significant enrichment of differential genes in the Flavonoid biosynthesis and Tropane, piperidine and pyridine alkaloid biosynthesis pathways under low phthalic acid treatment. The pathways that were significantly enriched under high concentration treatments were Glucosinolate biosynthesis and ABC transporters. In addition, MAG and 6-HSph were identified as core response metabolites under phthalic acid stress, key differential genes involved. Interestingly, *Pugionium cornutum* (L.) Gaertn. showed a more pronounced response to the stress of high concentrations of phthalic acid compared to the treatment with low concentrations of phthalic acid. This was evidenced by significant differential gene changes with increasing concentrations of autotoxic stress. Our findings provide the first evidence linking lipids to autotoxicity in *Pugionium cornutum* (L.) Gaertn. It provides a theoretical basis for further in-depth studies on the mechanisms of the effects of autotoxic substances on *Pugionium cornutum* (L.) Gaertn. and the mitigation of the continuous cropping disorder.

## Supporting information

S1 Table This is a table. Statistics on the number of metabolites. (DOCX)

S2 Table Statistics on the number of metabolites. (DOCX)

S1 Fig Supplementary figure. (PDF)
