# Supplementary material for: Integrative transcriptomic and metabolomic analyses provide insights into the mechanism of autotoxicity of Pugionium cornutum (L.) Gaertn
Source: PLoS One. 2025 Sep 17;20(9):e0331858. doi: 10.1371/journal.pone.0331858 (PMC12443292; doi:10.1371/journal.pone.0331858)
Supplement: S1 Table — (DOCX) [file pone.0331858.s001.docx]

| **Groups** | **All**  **metabolites** | **Upregulated**  **metabolites** | **Downregulated**  **metabolites** |
| --- | --- | --- | --- |
| Z1/CK | 457 | 223 | 234 |
| Z2/CK | 435 | 253 | 182 |

**Supplementary Table** **1.** This is a table. Statistics on the number of metabolites.
