# Supplementary material for: Integrative transcriptomic and metabolomic analyses provide insights into the mechanism of autotoxicity of Pugionium cornutum (L.) Gaertn
Source: PLoS One. 2025 Sep 17;20(9):e0331858. doi: 10.1371/journal.pone.0331858 (PMC12443292; doi:10.1371/journal.pone.0331858)
Supplement: S2 Table — (DOCX) [file pone.0331858.s002.docx]

| Sample | Raw Reads | Raw Bases | Clean Reads | Clean Bases | Valid Bases | Q30 | GC |
| --- | --- | --- | --- | --- | --- | --- | --- |
| CK | 46.17 | 6.92 | 45.67 | 6.63 | 95.66% | 96.17% | 45.31% |
| Z1 | 46.96 | 7.05 | 46.60 | 6.74 | 95.73% | 96.71% | 45.41% |
| Z2 | 47.23 | 7.08 | 46.77 | 6.76 | 95.41% | 96.32% | 45.64% |

Supplementary Table 2 Sample data quality control
