# Supplementary figures and images for: Integrative transcriptomic and metabolomic analyses provide insights into the mechanism of autotoxicity of Pugionium cornutum (L.) Gaertn
Source: PLoS One. 2025 Sep 17;20(9):e0331858. doi: 10.1371/journal.pone.0331858 (PMC12443292; doi:10.1371/journal.pone.0331858)

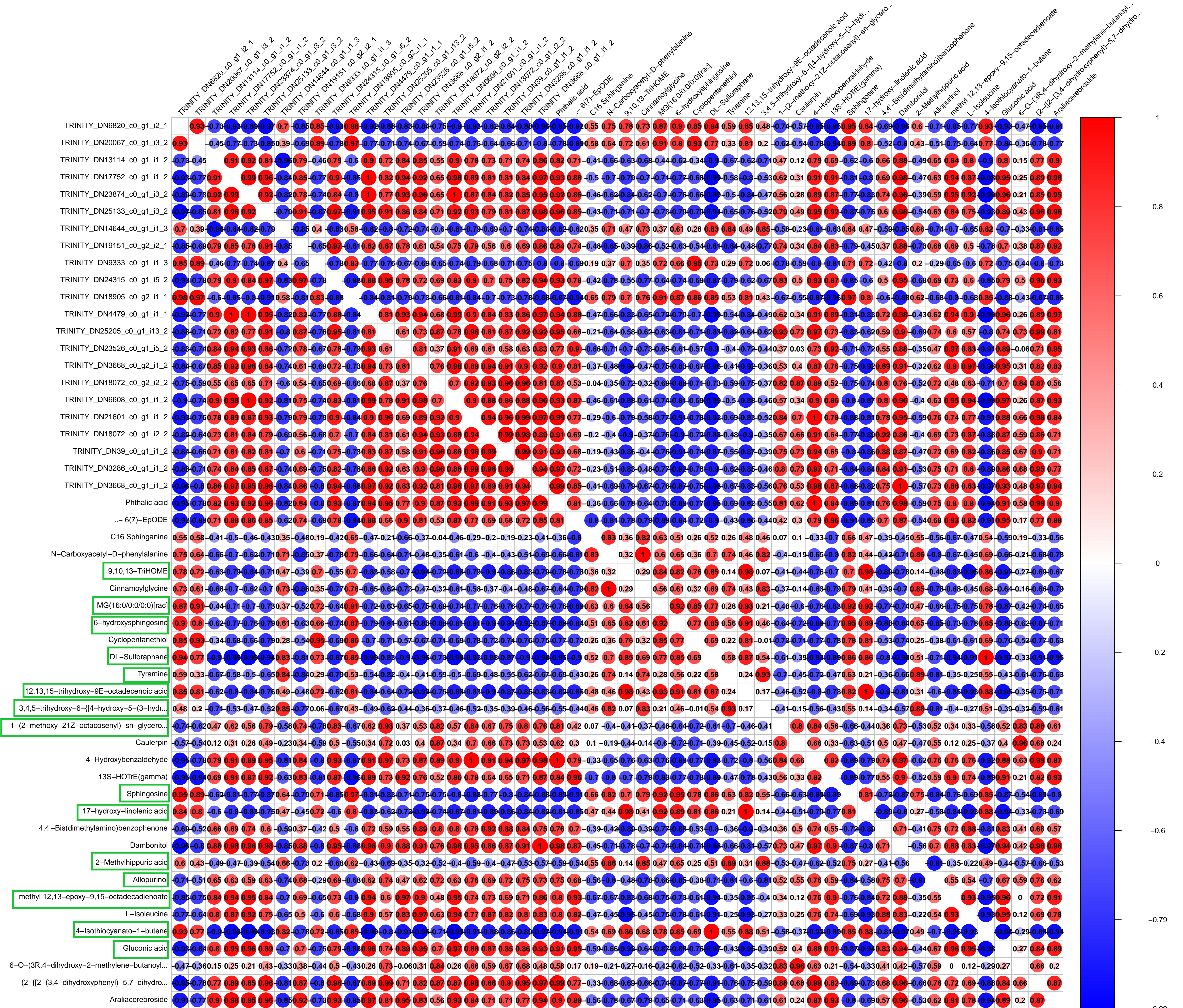

Supplement: S1 Fig — (PDF) [file pone.0331858.s003.pdf]
